#  Fatores associados à adesão a comportamentos preventivos da COVID-19 em participantes do ELSA-Brasil 

**DOI:** 10.1590/0102-311XPT188322

**Published:** 2023-10-09

**Authors:** Fernanda Garcia Gabira Miguez, Gabriela Oliveira, Oscar Geovanny Enriquez-Martinez, Maria de Jesus Mendes da Fonseca, Rosane Harter Griep, Sandhi Maria Barreto, Maria del Carmen Bisi Molina

**Affiliations:** 1 Universidade Federal do Espírito Santo, Vitória, Brasil.; 2 Escola Nacional de Saúde Pública Sergio Arouca, Fundação Oswaldo Cruz, Rio de Janeiro, Brasil.; 3 Instituto Oswaldo Cruz, Fundação Oswaldo Cruz, Rio de Janeiro, Brasil.; 4 Faculdade de Medicina, Universidade Federal de Minas Gerais, Belo Horizonte, Brasil.

**Keywords:** COVID-19, Pandemias, Isolamento Social, Comportamentos Relacionados com a Saúde, COVID-19, Pandemics, Social Isolation, Health Behavior, COVID-19, Pandemias, Aislamiento Social, Conductas Relacionadas con la Salud

## Abstract

O objetivo deste estudo foi avaliar a adesão a medidas de prevenção recomendadas durante a pandemia de COVID-19 e investigar os fatores associados a essa adesão na população adulta. Por meio de delineamento transversal, utilizam-se dados do estudo complementar *Estudo Longitudinal de Saúde do Adulto* (ELSA-Brasil) *- COVID*, realizado de 2020 a 2021, que foram analisados por meio do teste qui-quadrado e regressão logística multinomial. A amostra é composta por 5.440 participantes. A medida preventiva com maior adesão foi o uso de máscara facial (95,5%). Houve maior adesão pelo sexo feminino e menor chance de adesão pela raça/cor branca, por aqueles que consomem bebidas alcoólicas, aposentados, assim como para aqueles que moram sozinhos ou que possuem familiares que não seguiram as recomendações de ficar em casa. A maior adesão aos comportamentos preventivos foi verificada em apenas um terço da população participante, o que demonstra que havia a necessidade de uma maior conscientização quanto aos riscos em populações específicas. Os achados contribuem para melhorar o conhecimento sobre promoção da saúde e prevenção da COVID-19.

## Abstract (en)

The aim of the study is to assess the adherence to recommended prevention measures during the COVID-19 pandemic and to investigate the factors associated with this adherence in the adult population. This study has a cross-sectional design and used data from the complementary study *Brazilian Longitudinal Study of Adult Health* (ELSA-Brasil) *- COVID*, conducted from 2020 to 2021. Data were analyzed using the chi-square test and multinomial logistic regression. The sample consisted of 5,440 participants. The preventive measure with the highest adherence was the use of a face mask (95.5%). There was greater adherence by females and lower chance of adherence by white people, by those who consume alcoholic beverages, people who were retired, as well as for those who live alone or who have family members who did not follow the recommendations to stay at home. Greater adherence to preventive behaviors was observed in only one third of the participating population, which demonstrates that there was a need for greater awareness of the risks in specific populations. The findings contribute to improving our understanding about health promotion and COVID-19 prevention.

## Abstract (es)

El objetivo del estudio fue evaluar la adhesión a las medidas de prevención recomendadas durante la pandemia de COVID-19 e investigar los factores asociados con esta adhesión en la población adulta. El presente estudio tiene un diseño transversal y utilizó datos del estudio complementario *Estudio Longitudinal de Salud del Adulto en Brasil* (ELSA-Brasil) *- COVID*, realizado de 2020 a 2021. Los datos se analizaron mediante la prueba de chi-cuadrado y la regresión logística multinomial. La muestra fue compuesta por 5.440 participantes. La medida preventiva con mayor adhesión fue el uso de mascarilla facial (95,5%). Hubo mayor adhesión por el sexo femenino y menor posibilidad de adhesión por la raza/color blanca, por aquellos que consumen bebidas alcohólicas, jubilados, así como para aquellos que viven solos o que tienen familiares que no siguieron las recomendaciones de quedarse en casa. Solo se comprobó una mayor adhesión a los comportamientos preventivos en un tercio de la población participante, lo que demuestra que había la necesidad de una mayor concientización en cuanto a los riesgos en poblaciones específicas. Los resultados contribuyen a mejorar el conocimiento sobre la promoción de la salud y la prevención del COVID-19.

## Introdução

No ano de 2019, uma nova cepa de coronavírus (o SARS-CoV-2) foi relatada. A natureza infecciosa da doença, a mortalidade diária e sua capacidade de causar complicações graves em um período curto, como pneumonia aguda, síndrome do desconforto respiratório (SDR), insuficiência cardíaca, tempestade de citocinas e disfunção de múltiplos órgãos ^1^, ocasionou, até março de 2021, no Brasil, cerca de um terço de todas as mortes diárias por COVID-19 em todo o mundo ^2^.

Diante do cenário severo ocasionado pela pandemia, devido à ausência de tratamentos eficazes, o acesso e a disponibilidade limitada de vacinas durante o primeiro ano da pandemia, medidas de prevenção contra a COVID-19 foram estabelecidas para mitigar a propagação comunitária do COVID-19 em todo o território global, incluindo o isolamento de pacientes infectados ou suspeitos, uso de equipamentos de proteção individual (EPIs) como máscaras faciais, lavagem de mãos, uso de álcool em gel, distanciamento social, quarentenas e bloqueios obrigatórios ^3^.

Uma pesquisa evidenciou que o uso consistente de máscaras, lavagem das mãos e distanciamento físico são eficazes na prevenção da COVID-19 ^4^. Higienizar as mãos é uma das atividades mais significativas na interrupção da transmissão do vírus e sua propagação ^5^. Utilizar máscara adequadamente demonstrou ter associação significativa na redução do risco de infecções respiratórias em alguns estudos ^6^^,^^7^^,^^8^. Já no estudo de Álvarez-Pomar & Rojas-Galeano ^9^, o distanciamento físico é o hábito dominante na redução da disseminação de doenças.

A sustentabilidade e a efetividade destas medidas coexistiram com o estabelecimento de políticas de proteção social e apoio à população vulnerável durante as restrições da pandemia ^10^. No Brasil, em fevereiro de 2020, as medidas de enfrentamento à emergência do coronavírus foram sancionadas pela *Lei nº 13.979*, de 2020, dentre elas, a adoção de isolamento e quarentena ^11^. De acordo com Faria de Moura Villela et al. ^12^, no mesmo ano, no Brasil, a lavagem de mãos foi praticada por 98,7% dos 23.896 participantes do estudo. E destes, 92,6% aderiram à regra de distanciamento, mas apenas 45,5% usavam máscara facial ao sair - mesmo que posteriormente tenha ficado evidente que a dispersão de aerossóis em locais fechados e sem ventilação traziam mais riscos à população.

Alguns estudos mostram que os comportamentos de prevenção à COVID-19 podem ser influenciados pela compreensão de fatores sociodemográficos ^13^, idade ^14^, sexo ^12^^,^^15^, escolaridade ^12^^,^^15^^,^^16^, nível socioeconômico ^16^^,^^17^ e residir com outras pessoas ^15^.

Mesmo com o grande número de pesquisas brasileiras voltadas para o tema “COVID-19”, ainda há uma escassez de estudos que investigaram a adesão às medidas preventivas e os seus fatores relacionados. Nesse ínterim, verificar a adesão e entender como a prevenção foi influenciada por diferentes fatores dentro da amostra pertencente ao *Estudo Longitudinal de Saúde do Adulto* (ELSA-Brasil) ^10^ nos permitirá entender a adesão aos comportamentos preventivos realizados por essa população durante a pandemia. Assim, o objetivo deste estudo é avaliar a adesão a medidas de prevenção recomendadas durante a pandemia de COVID-19 e investigar os fatores associados a essa adesão na população adulta.

## Métodos

Foi executado um delineamento transversal e utilizam-se dados do estudo complementar aninhado ao ELSA-Brasil para avaliar os impactos de curto e longo prazo da COVID-19.

O ELSA-Brasil é uma coorte prospectiva composta por cinco universidades e um instituto de pesquisa de cidades brasileiras (Universidade Federal do Espírito Santo, Universidade Federal de Minas Gerais, Universidade Federal da Bahia, Universidade Federal de Rio Grande do Sul, Universidade Federal de São Paulo e Fundação Oswaldo Cruz), iniciada em agosto de 2008, com funcionários ativos ou aposentados, com idade entre 35 e 74 anos ^18^.

As avaliações de linha de base ocorreram em 2008-2010 e incluíram 15.105 participantes com 35 a 74 anos de idade que foram submetidos a exames clínicos e entrevistas. A primeira onda de acompanhamento ocorreu em 2012-2014 com amostra final de 14.014 e a segunda onda de seguimento nos anos de 2017 a 2019 com amostra de 12.636, sem recrutamento de novos participantes ^10^^,^^18^^,^^19^. Em cada onda, as avaliações consistiram em entrevistas, exames clínicos, coleta sobre dados sociodemográficos, histórico clínico pessoal e familiar de doenças, estilo de vida, medidas antropométricas e exames laboratoriais ^10^^,^^18^^,^^19^.

De junho de 2020 a março de 2021, os participantes da segunda onda de acompanhamento (n = 12.636), exceto os de São Paulo (n = 4.194), foram convidados a participar do estudo complementar sobre dados da COVID-19, de forma que 5.639 participantes (66.79%) aceitaram, assinaram o Termo de Consentimento Livre e Esclarecido e responderam aos questionários. Destes, utilizamos dados de 5.440 (97,46%), pois foram excluídos aqueles que não responderam sobre as variáveis de interesse.

Os dados do estudo complementar foram coletados *online* pelo celular ou computador, por meio de um aplicativo desenvolvido especialmente para o estudo, ou por contato telefônico com auxílio de um profissional treinado e equipe certificada. O estudo foi aprovado pelos comitês de ética de todos os centros de pesquisa do ELSA-Brasil e pelo Comitê Nacional de Ética em Pesquisa (CONEP; nº 13.065; CAAE: 0016,1,198,000-06).

O questionário do “ELSA-Brasil COVID” foi dividido em quatro módulos para a aplicação, foram feitas perguntas contidas em três dos quatro módulos: Módulo I (adesão ao distanciamento social e exposição; peso; sono; álcool), Módulo II (história ocupacional) e Módulo III (impacto na renda).

Para verificar a adesão aos comportamentos preventivos foi realizada a construção de um escore a partir de seis perguntas contidas no Módulo I: “lavar as mãos com água e sabão por 20 segundos; usar álcool 70% gel/líquido nas mãos; cobrir o nariz e a boca ao tossir e espirrar; retirar os sapatos antes de entrar em casa; trocar de roupa ao chegar em casa; usar máscara sempre que sair de casa; lavar as embalagens dos produtos de mercado ou farmácia antes de guardá-los; não cumprimentar as pessoas com beijo no rosto ou aperto de mãos”. Com cinco opções de resposta, categorizadas em dados contínuos: sempre = 5, quase sempre = 4, às vezes = 3, raramente = 2 e nunca = 1 (escala de Likert); obtendo um escore com variabilidade de 5 a 30 pontos e categorizada em tercil.

Além disso, foram utilizados dados sociodemográficos para caracterização da população, como sexo, idade, raça/cor (branca; não branca), situação atual de trabalho (ativo; aposentado, mas continua trabalhando; aposentado e não está trabalhando) e estado civil (casado/união estável; separado/divorciado; viúvo(a); solteiro(a)), também foi perguntado se houve mudança na renda durante a pandemia (sim; não). Variáveis de hábitos de vida também foram aferidas, através das seguintes perguntas: “Nas últimas trinta noites com que frequência teve dificuldade em pegar no sono?” (nunca; raramente; às vezes; sempre); “Desde o início do distanciamento social você consumiu algum tipo de bebida alcoólica?” (não; sim); “Fuma cigarros atualmente?” (nunca fumou; fumou, mas parou; fuma); “Você percebeu alguma alteração de peso ou de medidas corporais durante o período de distanciamento social?” (não, mantive meu peso; sim, perdi peso; sim, ganhei peso).

As análises estatísticas foram todas realizadas no Stata 16.0 (https://www.stata.com). As variáveis categóricas foram analisadas por meio do teste estatístico qui-quadrado para verificar a distribuição das variáveis de exposição à adesão de comportamentos preventivos pelos participantes da pesquisa. Para analisar os fatores associados ao desfecho, foi realizada regressão logística multinominal em cada tercil de adesão, da seguinte forma: modelo bruto, após ajustado por modelo 1: variáveis sociodemográficas (sexo, faixa etária, raça/cor, situação atual de trabalho e estado civil); modelo 2: modelo 1 + hábitos de vida (sono, fumo, peso e bebida) e modelo 3: modelo 1 + modelo 2 + variáveis de recomendação de isolamento social (recomendação de ficar em casa, recomendação de ficar em casa pelos familiares e quantos dias da semana saiu de casa). Adotou-se em todas as análises estatísticas, nível de significância de 5%.

## Resultados

O presente estudo é composto por 5.440 participantes que responderam às perguntas de interesse, desses, 36% referiram maior adesão, 33,7% menor adesão e 30,2% adesão intermediária aos comportamentos preventivos contra a COVID-19. Conforme apresentado na Tabela 1, há maior adesão às medidas preventivas por participantes do sexo feminino, não brancos, de 53 a 63 anos, ativos laboralmente, casados ou em união estável, que sofreram mudança na renda familiar, que nos últimos 30 dias raramente tiveram dificuldade de pegar no sono, nunca fumaram, ganharam peso, consomem bebida alcoólica, que seguiram as recomendações de ficar em casa, assim como seus familiares, que saíram de 2 a 4 dias por semana, que fizeram isolamento social devido o contato com alguém com COVID-19, e não realizaram isolamento devido a algum sintoma gripal.

**Tabela 1:** Fatores sociodemográficos, hábitos de vida e realização de quarentena em relação a adesão de comportamentos preventivos por participantes do ELSA-Brasil COVID. *Estudo Longitudinal de Saúde do Adulto*, 2020-2021.

Características	Escore de proteção				Valor de p
	Total (%)	Mais adesão (%)	Adesão intermediária (%)	Menos adesão (%)	
Total	5.440 (100,0)	1.960 (36,0)	1.645 (30,2)	1.835 (33,7)	
Sexo					
Feminino	3.131 (57,6)	1.270 (40,5)	998 (31,8)	863 (27,5)	< 0,001
Masculino	2.309 (42,4)	690 (29,8)	647 (28,0)	972 (42,1)	
Faixa etária (anos)					
42-52	1.295 (24,3)	469 (36,2)	363 (30,3)	433 (33,4)	
53-63	2.422 (45,5)	905 (37,3)	747 (30,8)	770 (31,7)	< 0,001
64-74	1.345 (25,3)	461 (34,2)	409 (30,4)	475 (35,3)	
75-84	253 (4,7)	72 (28,4)	58 (22,9)	123 (48,6)	
Raça/Cor					
Não branco	2.334 (44,3)	946 (40,5)	680 (21,1)	708 (30,3)	
Branco	2.927 (55,6)	941 (32,1)	908 (31,0)	1.078 (36,8)	< 0,001
Trabalho					
Ativo	2.445 (48,4)	916 (37,4)	747 (30,5)	782 (31,9)	
Aposentado, mas continua trabalhando	544 (10,7)	180 (33,0)	166 (30,5)	198 (36,4)	< 0,001
Aposentado e não está trabalhando	2.055 (40,7)	704 (34,2)	621 (30,2)	730 (35,5)	
Estado civil					
Casado/União estável	3.317 (62,4)	1.149 (34,6)	1.025 (30,9)	1.143 (34,4)	
Separado/Divorciado	893 (16,8)	341 (38,1)	252 (28,2)	300 (33,5)	< 0,043
Viúvo(a)	267 (05,0)	84 (31,4)	86 (32,2)	97 (36,3)	
Solteiro(a)	836 (15,7)	333 (39,8)	243 (29,0)	260 (31,1)	
Mudança da renda					
Não	1.965 (45,2)	700 (35,6)	586 (29,8)	679 (34,5)	
Sim	2.382 (54,8)	863 (36,2)	747 (31,6)	772 (32,4)	< 0,297
Dificuldade para dormir					
Nunca	1.033 (20,6)	367 (35,5)	284 (27,4)	382 (36,9)	
Raramente	1.710 (34,1)	575 (33,6)	530 (30,9)	605 (35,3)	
Às vezes	1.433 (28,6)	532 (37,1)	439 (30,6)	462 (32,2)	< 0,004
Sempre	830 (16,6)	326 (39,2)	260 (31,3)	244 (29,4)	
Fumo					
Nunca fumou	3.246 (64,8)	1.219 (37,5)	996 (30,6)	1.031 (31,7)	
Fumava, mas parou	1.437 (28,7)	476 (33,1)	429 (29,8)	532 (37,0)	< 0,000
Fuma	323 (6,4)	105 (32,5)	88 (27,2)	130 (40,2)	
Peso					
Não, mantive meu peso	1.888 (37,7)	637 (33,7)	558 (29,5)	693 (36,7)	
Sim, perdi peso	910 (18,2)	378 (41,5)	266 (29,2)	266 (29,2)	< 0,000
Sim, ganhei peso	2.208 (44,1)	785 (33,5)	689 (31,2)	734 (33,2)	
Bebida alcoólica					
Não	1.934 (38,6)	795 (41,1)	531 (27,4)	608 (35,9)	< 0,000
Sim	3.072 (61,3)	1.005 (32,8)	982 (31,9)	1.085 (35,3)	
Recomendação de ficar em casa					
Sim	4.534 (83,3)	1.656 (36,5)	1.410 (31,1)	1.468 (32,3)	< 0,000
Não	906 (16,6)	304 (33,5)	235 (25,9)	367 (40,5)	
Recomendação de ficar em casa por familiares					
Sim	3.914 (71,9)	1.507 (38,5)	1.197 (30,5)	1.210 (30,9)	
Não	756 (13,9)	200 (26,4)	216 (28,5)	340 (44,9)	< 0,000
Moro sozinho	770 (14,1)	253 (32,8)	232 (30,1)	285 (37,0)	
Quantos dias saiu por semana					
≤ 1	1.943 (35,8)	832 (42,8)	610 (31,3)	501 (25,7)	< 0,000
2-4	2.447 (45,0)	821 (33,5)	762 (31,1)	864 (35,3)	
5-7	1.039 (19,1)	303 (29,1)	268 (25,7)	468 (45,0)	
Isolamento por contato com alguém com COVID-19					
Não	4.374 (80,4)	1.572 (35,9)	1.312 (30,0)	1.490 (34,0)	< 0,543
Sim	1.066 (19,6)	388 (36,4)	333 (31,2)	345 (32,3)	
Isolamento devido sintoma gripal					
Não	4.709 (86,5)	1.676 (35,5)	1.421 (30,1)	1.612 (34,2)	
Sim	731 (13,4)	284 (38,8)	224 (30,6)	223 (30,5)	< 0,105

IC95%: intervalo de 95% de confiança; OR: *odds ratio*.

**Figura 1:**
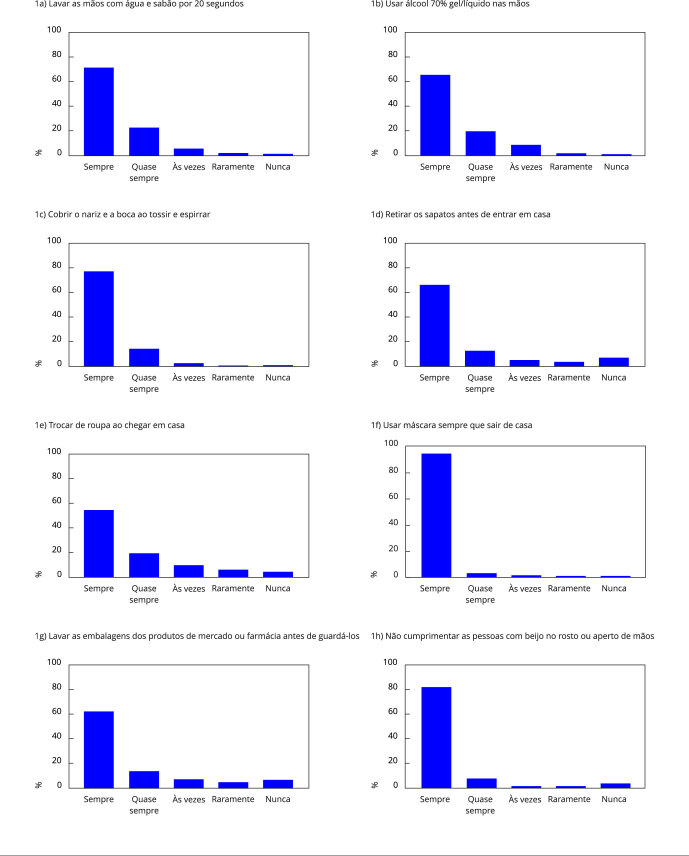
Proporção da adesão aos comportamentos preventivos por participantes do ELSA-Brasil COVID. *Estudo Longitudinal de Saúde do Adulto*, 2020-2021.

Dentre as medidas preventivas avaliadas, na categoria “sempre”, “usar máscara sempre que sair de casa” foi a mais prevalente (95,5%), seguida de “não cumprimentar as pessoas com beijo no rosto ou aperto de mãos” (83,7%), já “trocar de roupa ao chegar em casa” (56%) foi a medida menos realizada (Figura 1).

De acordo com a Tabela 2, referente ao grupo com mais adesão aos comportamentos preventivos, após os ajustes da análise, ser do sexo feminino e ter relato de perda de peso tiveram mais chance de aderirem a maior parte dos comportamentos preventivos durante a primeira onda da pandemia.

Quanto a Tabela 3, para o grupo de pessoas com adesão intermediária, assim como na maior adesão, ser do sexo feminino aumentou em 26% a chance de adesão aos comportamentos preventivos à COVID-19. A adesão intermediária foi maior também naqueles que referiram ser consumidores de bebidas alcoólicas.

**Tabela 2:** Comparação dos fatores sociodemográficos, hábitos de vida e confinamento em relação à maior adesão aos comportamentos preventivos comparados à adesão intermediária e a menor adesão por participantes do ELSA-Brasil COVID. *Estudo Longitudinal de Saúde do Adulto*, 2020-2021.

Características	OR bruto (IC95%)	Modelo 1	Modelo 2	Modelo 3
OR (IC95%)	OR (IC95%)	OR (IC95%)
Sexo				
Feminino	1,60 (1,40-1,70)	1,67 (1,40-1,90)	1,60 (1,40-1,80)	1,56 (1,30-1,80)
Masculino	1,00	1,00	1,00	1,00
Faixa etária (anos)				
42-52	1,00	1,00	1,00	1,00
53-63	1,05 (0,90-1,20)	1,11 (0,90-1,30)	1,14 (0,90-1,30)	1,12 (0,90-1,30)
64-74	0,91 (0,70-1,00)	1,07 (0,80-1,30)	1,10 (0,90-1,30)	1,05 (0,80-1,30)
75-84	0,70 (0,50-0,90)	0,90 (0,60-1,20)	0,97 (0,60-1,40)	0,86 (0,60-1,20)
Raça/Cor				
Não branco	1,00	1,00	1,00	1,00
Branco	0,69 (0,60-0,70)	0,72 (0,60-0,80)	0,75 (0,60-0,80)	0,74 (0,60-0,80)
Trabalho				
Continua ativo	1,00	1,00	1,00	1,00
Aposentado, mas continua trabalhando	0,82 (0,60-1,00)	0,82 (0,60-1,00)	0,78 (0,60-0,90)	0,80 (0,60-1,00)
Aposentado e não está trabalhando	0,86 (0,70-0,90)	0,80 (0,70-0,90)	0,78 (0,60-0,90)	0,76 (0,60-0,90)
Estado civil				
Casado/União estável	1,00	1,00	1,00	1,00
Separado/Divorciado	1,16 (1,00-1,30)	1,01 (0,80-1,20)	1,02 (0,80-1,20)	1,09 (0,90-1,30)
Viúvo(a)	0,86 (0,60-1,10)	0,75 (0,50-1,00)	0,73 (0,50-1,00)	0,74 (0,50-1,00)
Solteiro(a)	1,24 (1,00-1,40)	0,98 (0,80-1,10)	1,01 (0,80-1,20)	1,09 (0,90-1,30)
Dificuldade para dormir				
Nunca	1,00	-	1,00	1,00
Raramente	0,91 (0,70-1,00)	-	0,89 (0,70-1,00)	0,89 (0,70-1,00)
Às vezes	1,07 (0,90-1,20)	-	0,99 (0,80-1,20)	1,00 (0,80-1,20)
Sempre	1,17 (0,90-1,40)		1,04 (0,80-1,30)	1,04 (0,80-1,30)
Fumo				
Nunca fumou	1,00	-	1,00	1,00
Fumava, mas parou	0,82 (0,70-0,90)	-	0,92 (0,80-1,00)	0,92 (0,80-1,00)
Fuma	0,80 (0,60-1,00)	-	0,78 (0,60-1,00)	0,80 (0,60-1,00)
Peso				
Não, mantive meu peso	1,00	-	1,00	1,00
Sim, perdi peso	1,39 (1,10-1,60)	-	1,35 (1,10-1,60)	1,33 (1,10-1,60)
Sim, ganhei peso	1,08 (0,90-1,20)	-	1,05 (0,90-1,20)	1,05 (0,90-1,20)
Bebida alcoólica				
Sim	0,69 (0,60-0,70)	-	0,75 (0,60-0,80)	0,76 (0,60-0,80)
Não	1,00	-	1,00	1,00
Recomendação de ficar em casa				
Sim	1,00	-	-	1,00
Não	0,87 (0,70-1,00)	-	-	1,05 (0,80-1,30)
Recomendação de ficar em casa por familiares				
Sim	1,00		-	1,00
Não	0,57 (0,40-0,60)	-	-	0,58 (0,40-0,70)
Moro sozinho	0,78 (0,60-0,90)	-	-	0,73 (0,60-0,90)
Quantos dias saiu por semana				
≤ 1	1,00	-	-	1,00
2-4	0,67 (0,50-0,70)	-	-	0,72 (0,60-0,80)
5-7	0,64 (0,40-0,60)	-	-	0,62 (0,50-0,70)

IC95%: intervalo de 95% de confiança; OR: *odds ratio*.

**Tabela 3:** Comparação dos fatores sociodemográficos, hábitos de vida e confinamento em relação à adesão intermediária aos comportamentos preventivos comparado à maior e menor adesão por participantes do ELSA-Brasil COVID. *Estudo Longitudinal de Saúde do Adulto*, 2020-2021.

Características	OR bruto (IC95%)	Modelo 1	Modelo 2	Modelo 3
OR (IC95%)	OR (IC95%)	OR (IC95%)
Sexo				
Feminino	1,20 (1,00-1,30)	1,23 (1,10-1,40)	1,28 (1,10-1,50)	1,26 (1,10-1,40)
Masculino	1,00	1,00	1,00	1,00
Faixa etária (anos)				
42-52	1,00	1,00	1,00	1,00
53-63	1,02 (0,80-1,10)	1,06 (0,90-1,20)	1,09 (0,90-1,30)	1,07 (0,90-1,30)
64-74	1,00 (0,80-1,10)	1,04 (0,80-1,30)	1,07 (0,80-1,30)	1,02 (0,80-1,30)
75-84	0,68 (0,40-0,90)	0,72 (0,50-1,00)	0,71 (0,50-1,00)	0,67 (0,40-1,00)
Raça/Cor				
Não branco	1,00	1,00	1,00	1,00
Branco	1,09 (0,90-1,20)	1,11 (0,90-1,20)	1,09 (0,90-1,20)	1,08 (0,90-1,20)
Trabalho				
Continua ativo	1,00	1,00	1,00	1,00
Aposentado, mas continua trabalhando	0,99 (0,80-1,20)	0,97 (0,70-1,20)	0,98 (0,80-1,20)	1,01 (0,80-1,20)
Aposentado e não está trabalhando	0,98 (0,80-1,10)	0,94 (0,80-1,10)	0,93 (0,80-1,10)	0,90 (0,70-1,00)
Estado civil				
Casado/União estável	1,00	1,00	1,00	1,00
Separado/Divorciado	0,87 (0,70-1,00)	0,81 (0,70-0,90)	0,81 (0,70-1,00)	0,79 (0,60-0,90)
Viúvo(a)	1,06 (0,80-1,30)	1,03 (0,70-1,40)	1,11 (0,80-1,50)	1,08 (0,80-1,40)
Solteiro(a)	0,91 (0,70-1,00)	0,87 (0,70-1,00)	0,86 (0,70-1,00)	0,83 (0,70-1,00)
Dificuldade para dormir				
Nunca	1,00	-	1,00	1,00
Raramente	1,18 (0,90-1,40)	-	1,17 (0,90-1,40)	1,17 (0,90-1,40)
Às vezes	1,16 (0,90-1,30)	-	1,11 (0,90-1,30)	1,11 (0,90-1,30)
Sempre	1,20 (0,90-1,40)		1,13 (0,90-1,40)	1,14 (0,90-1,40)
Fumo				
Nunca fumou	1,00	-	1,00	1,00
Fumava, mas parou	0,96 (0,80-1,10)	-	0,96 (0,80-1,10)	0,95 (0,80-1,10)
Fuma	0,84 (0,60-1,00)	-	0,77 (0,60-1,00)	0,78 (0,60-1,00)
Peso				
Não, mantive meu peso	1,00	-	1,00	1,00
Sim, perdi peso	0,98 (0,80-1,10)	-	0,93 (0,70-1,10)	0,90 (0,70-1,10)
Sim, ganhei peso	1,08 (0,90-1,20)	-	0,99 (0,80-1,10)	0,99 (0,80-1,10)
Bebida alcoólica				
Sim	1,24 (1,00-1,40)	-	1,31 (1,10-1,50)	1,30 (1,10-1,50)
Não	1,00	-	1,00	1,00
Recomendação de ficar em casa				
Sim	1,00	-	-	1,00
Não	0,77 (0,60-0,90)	-	-	0,90 (0,70-1,10)
Recomendação de ficar em casa por familiares				
Sim	1,00	-	-	1,00
Não	0,90 (0,70-1,00)	-	-	0,90 (0,70-1,10)
Moro sozinho	0,97 (0,80-1,10)	-	-	1,08 (0,90-1,30)
Quantos dias saiu por semana				
≤ 1	1,00	-	-	1,00
2-4	0,98 (0,80-1,10)	-	-	0,96 (0,80-1,10)
5-7	0,75 (0,60-0,80)	-	-	0,78 (0,60-0,90)

IC95%: intervalo de 95% de confiança; OR: *odds ratio*.

Já na Tabela 4, referente a menor chance de adesão dos indivíduos do estudo, ser do sexo feminino reduz a chance de adotar menos medidas preventivas. Os participantes brancos tiveram 26% mais chance de não adotarem medidas preventivas, assim como participantes aposentados e que não estão trabalhando, o mesmo ocorreu para aqueles que possuem familiares que não estão seguindo as recomendações de ficar em casa e para quem está saindo mais vezes de casa durante a semana.

**Tabela 4:** Comparação dos fatores sociodemográficos, hábitos de vida e confinamento em relação à menor adesão aos comportamentos preventivos comparados à adesão intermediária e maior adesão por participantes do ELSA-Brasil COVID. *Estudo Longitudinal de Saúde do Adulto*, 2020-2021.

Características	OR bruto (IC95%)	Modelo 1	Modelo 2	Modelo 3
OR (IC95%)	OR (IC95%)	OR (IC95%)
Sexo				
Feminino	0,52 (0,40-0,50)	0,48 (0,40-0,50)	0,49 (0,40-0,50)	0,50 (0,40-0,60)
Masculino	1,00	1,00	1,00	1,00
Faixa etária (anos)				
42-52	1,00	1,00	1,00	1,00
53-63	0,92 (0,80-1,00)	0,83 (0,70-0,90)	0,79 (0,60-0,90)	0,82 (0,70-1,00)
64-74	1,08 (0,90-1,20)	0,88 (0,70-1,10)	0,83 (0,60-1,00)	0,90 (0,70-1,10)
75-84	1,88 (1,40-2,40)	1,37 (0,90-1,90)	1,26 (0,90-1,80)	1,5 (1,20-2,20)
Raça/Cor				
Não branco	1,00	1,00	1,00	1,00
Branco	1,33 (1,10-1,50)	1,26 (1,10-1,40)	1,23 (1,10-1,40)	1,26 (1,10-1,40)
Trabalho				
Continua ativo	1,00	1,00	1,00	1,00
Aposentado, mas continua trabalhando	1,21 (1,00-1,40)	1,25 (1,00-1,50)	1,31 (1,00-1,60)	1,24 (0,90-1,50)
Aposentado e não está trabalhando	1,17 (1,00-1,30)	1,33 (1,10-1,50)	1,38 (1,10-1,60)	1,46 (1,20-1,70)
Estado civil				
Casado/União estável	1,00	1,00	1,00	1,00
Separado/Divorciado	0,96 (0,80-1,10)	1,20 (1,00-1,40)	1,18 (0,90-1,40)	1,12 (0,90-1,30)
Viúvo(a)	1,08 (0,80-1,40)	1,30 (0,90-1,70)	1,25 (0,90-1,70)	1,24 (0,90-1,70)
Solteiro(a)	0,85 (0,70-1,00)	1,15 (0,90-1,30)	1,13 (0,90-1,30)	1,07 (0,80-1,30)
Dificuldade para dormir				
Nunca	1,00	-	1,00	1,00
Raramente	0,93 (0,70-1,00)	-	0,96 (0,80-1,10)	0,96 (0,80-1,10)
Às vezes	0,81 (0,60-0,90)	-	0,91 (0,70-1,10)	0,89 (0,70-1,00)
Sempre	0,70 (0,50-0,80)		0,84 (0,70-1,00)	0,82 (0,60-1,00)
Fumo				
Nunca fumou	1,00	-	1,00	1,00
Fumava, mas parou	1,26 (1,10-1,40)	-	1,12 (0,90-1,30)	1,12 (0,90-1,30)
Fuma	1,44 (1,10-1,80)	-	1,58 (1,20-2,00)	1,53 (1,00-1,30)
Peso				
Não, mantive meu peso	1,00	-	1,00	1,00
Sim, perdi peso	0,71 (0,60-0,80)	-	0,77 (0,60-0,90)	0,80 (0,60-0,90)
Sim, ganhei peso	0,85 (0,70-0,90)	-	0,95 (0,80-1,10)	0,95 (0,80-1,10)
Bebida alcoólica				
Sim	1,19 (1,00-1,30)	-	1,03 (0,90-1,20)	1,01 (0,80-1,10)
Não	1,00	-	1,00	1,00
Recomendação de ficar em casa				
Sim	1,00	-	-	1,00
Não	1,42 (1,20-1,60)	-	-	1,06 (0,80-1,30)
Recomendação de ficar em casa por familiares				
Sim	1,00	-	-	1,00
Não	1,82 (1,50-2,10)	-	-	1,84 (1,50-2,20)
Moro sozinho	1,31 (1,10-1,50)	-	-	1,28 (1,00-1,50)
Quantos dias saiu por semana				
≤ 1	1,00	-	-	1,00
2-4	1,57 (1,30-1,70)	-	-	1,48 (1,30-1,70)
5-7	2,35 (2,00-2,70)	-	-	2,00 (1,60-2,40)

IC95%: intervalo de 95% de confiança; OR: *odds ratio*.

## Discussão

A pesquisa fornece dados importantes sobre as variáveis associadas à adesão dos comportamentos preventivos dos participantes do ELSA-Brasil COVID durante a pandemia da COVID-19.

Nossos resultados evidenciam que ser mulher é um fator positivamente associado à presença de comportamentos preventivos, sendo consistente com estudos realizados em outras partes do mundo ^20^^,^^21^. Mulheres são mais propensas à conscientização em relação à saúde e à prevenção de doenças, além de terem uma melhor percepção de riscos ^22^. Como estão mais envolvidas com a preparação de refeições e cuidados com os filhos, tendem a aderir mais a medidas preventivas em relação a doenças infecciosas comparado ao sexo masculino ^23^, visto que há uma associação entre uma maior percepção de risco e adesão às medidas preventivas ^24^.

O relato de perda de peso também esteve associado a uma maior adesão, a perda de peso pode ter sido influenciada pela apreensão e incerteza sobre situação de saúde global nessa população, levando a modificações comportamentais e no estilo de vida e alimentação. Além do mais, já é conhecido que o bem-estar emocional pode afetar o apetite ^25^. Estudos também verificaram uma redução no consumo de *fast food* e aumento na ingestão de refeições caseiras ^26^^,^^27^, uma dieta menos inflamatória ^28^ e mudanças para um padrão mais saudável ^29^ durante a pandemia, o que contribui para uma alimentação mais saudável e redução de peso. Ainda, uma metanálise avaliou o impacto do bloqueio causado pela pandemia no peso corporal da população em geral, evidenciando perda significativa ^26^.

Identificou-se que ser aposentado e não exercer outra função empregatícia teve maior propensão a não seguir as recomendações de adesão preventiva, isso também é visto para aqueles que relataram sair mais vezes de casa durante a semana. De acordo com Shati et al. ^30^, idosos, uma população majoritariamente aposentada, que residiam sozinhos necessitavam sair mais de suas casas para suprir suas próprias necessidades diárias, tendo em vista a redução de visitas que auxiliavam nesse apoio. Isto corrobora achados de Bearth et al. ^31^, por exemplo, as motivações para essa população sair de casa são: sensação de independência e poder comprar os próprios mantimentos.

De acordo com Flett & Heisel ^32^, o distanciamento social rigoroso pode ser uma medida desafiadora para pessoas mais velhas. Sabe-se que, no começo da pandemia, iniciativas foram tomadas para auxiliar no cotidiano das pessoas, contudo, conforme explicam Bearth et al. ^31^, nem toda a população mais velha pode se beneficiar dessas iniciativas, às vezes por desconhecimento, por recusa ou pela localidade da residência.

Em estudo realizado com sul-asiáticos, o conhecimento inadequado sobre a COVID-19 esteve associado a menor adesão a atitudes relacionadas à prevenção ^33^. De acordo com Baek et al. ^34^, canais e fontes de informação de confiança têm efeitos nos comportamentos preventivos e, se há mais confiança nas informações divulgadas pelo governo, há maior frequência nas práticas dos comportamentos preventivos.

Estudos que analisam a associação entre a adoção de medidas preventivas à COVID-19 e variáveis sobre comportamento em saúde, como o uso de bebidas alcóolicas, foram menos explorados na literatura. Em nosso estudo, ser consumidor de bebidas alcoólicas associa-se à redução em 76% na realização de comportamentos preventivos durante a pandemia comparado a quem não fez uso de bebidas alcoólicas. Tal fato foi verificado em outros estudos ^35^^,^^36^^,^^37^, indivíduos que faziam uso de bebida alcoólica diariamente relataram menor adesão aos comportamentos preventivos quando comparados àqueles que não bebiam diariamente ^35^.

O isolamento e o estresse vêm sendo sugeridos na literatura como fatores significativos para o consumo de bebidas alcoólicas ^38^^,^^39^. A presença de hábitos não saudáveis pode prever comportamentos menos conscientes da saúde durante uma pandemia, contribuindo para o não cumprimento das medidas de proteção.

A coorte do ELSA-Brasil é bem definida, o que aumenta a validade externa e a generalização dos dados, contudo, este estudo apresenta limitações, pois sua amostragem é ocupacional e não populacional, visto que é composta por servidores públicos de universidades participantes da pesquisa, que possuem renda média salarial superior a nacional. Além disso, os participantes desta pesquisa realizaram o preenchimento dos questionários de forma voluntária, o que pode gerar um viés de seleção e influenciar as informações sobre a adesão às medidas de prevenção. Tal limitação também foi observada em outros estudos realizados durante o período pandêmico com amostra de conveniência e obedecem ao distanciamento social recomendado ^30^^,^^36^. Dessa maneira, os resultados não podem ser inferidos nacionalmente, mas sim interpretados dentro do contexto da amostra, representando uma parte da população brasileira com características semelhantes.

Apesar disso, o este estudo inova ao verificar a adesão aos comportamentos preventivos recomendados pelas instituições de saúde, trazendo à luz informações importantes e pouco investigadas que podem contribuir para melhor esclarecimento sobre o tema e as variáveis correlacionadas.

A maioria dos estudos ^40^^,^^41^ categorizam a adesão às práticas comportamentais de uso de medidas preventivas contra COVID-19 em maior e menor adesão. No entanto, essa abordagem pode ser analisada de forma mais complexa, considerando que existem diferentes tipos de medidas protetivas, algumas das quais só são possíveis para aqueles com maior poder aquisitivo. Classificar essa variável em apenas dois grupos pode não ser a melhor escolha, pois pode mascarar pessoas que adotam algumas ações contra a COVID-19, mas não todas, devido a diferentes aspectos específicos. Além disso, a economia familiar foi identificada como um fator importante para a aderência às medidas de proteção. Pessoas que percebem os custos a longo prazo podem evitar medidas que exigem gastos econômicos e optar por outras que não os tenham ^42^.

O estudo foi realizado de junho de 2020 a março de 2021, quando o governo brasileiro já havia sancionado a *Lei nº 13.979/2020*^11^, que dispõe sobre as medidas que poderiam ser adotadas para enfrentamento da pandemia. Contudo, o próprio presidente do país gerou muita confusão em como lidar com a COVID-19, houve negligência em relação a sua gravidade ^43^, além da demora na implantação de medidas de saúde pública. Esse cenário resultou em um conflito político somado à crise sanitária no país, influenciando a implementação das medidas de controle ^10^.

Por fim, apenas um terço da população analisada apresentou uma maior adesão aos comportamentos preventivos para essa doença que mobilizou a ciência e saúde de diferentes países. A negligência e demora na implementação de medidas pelas instituições brasileiras podem ter afetado, e muito, a adesão da população às medidas preventivas.
